# Effectiveness of prolonged use of continuous passive motion (CPM), as an adjunct to physiotherapy, after total knee arthroplasty

**DOI:** 10.1186/1471-2474-9-60

**Published:** 2008-04-29

**Authors:** Ton AF Lenssen, Mike JA van Steyn, Yvonne HF Crijns, Eddie MH Waltjé, George M Roox, Ruud JT Geesink, Piet A van den Brandt, Rob A De Bie

**Affiliations:** 1 University Hospital Maastricht, Department of Physiotherapy, Maastricht, The Netherlands; 2 University Hospital Maastricht, Department of Orthopaedics, Maastricht, The Netherlands; 3 Maastricht University, Department of Epidemiology, Maastricht, The Netherlands

## Abstract

**Background:**

Adequate and intensive rehabilitation is an important requirement for successful total knee arthroplasty.

Although research suggests that Continuous Passive Motion (CPM) should be implemented in the first rehabilitation phase after surgery, there is substantial debate about the duration of each session and the total period of CPM application. A Cochrane review on this topic concluded that short-term use of CPM leads to greater short-term range of motion. It also suggested, however, that future research should concentrate on the treatment period during which CPM should be administered.

**Methods:**

In a randomised controlled trial we investigated the effectiveness of prolonged CPM use in the home situation as an adjunct to standardised PT. Efficacy was assessed in terms of faster improvements in range of motion (RoM) and functional recovery, measured at the end of the active treatment period, 17 days after surgery.

Sixty patients with knee osteoarthritis undergoing TKA and experiencing early postoperative flexion impairment were randomised over two treatment groups. The experimental group received CPM + PT for 17 consecutive days after surgery, whereas the usual care group received the same treatment during the in-hospital phase (i.e. about four days), followed by PT alone (usual care) in the first two weeks after hospital discharge.

From 18 days to three months after surgery, both groups received standardised PT. The primary focus of rehabilitation was functional recovery (e.g. ambulation) and regaining RoM in the knee.

**Results:**

Prolonged use of CPM slightly improved short-term RoM in patients with limited RoM at the time of discharge after total knee arthroplasty when added to a semi-standard PT programme. Assessment at 6 weeks and three months after surgery found no long-term effects of this intervention Neither did we detect functional benefits of the improved RoM at any of the outcome assessments.

**Conclusion:**

Although results indicate that prolonged CPM use might have a small short-term effect on RoM, routine use of prolonged CPM in patients with limited RoM at hospital discharge should be reconsidered, since neither long-term effects nor transfer to better functional performance was detected.

**Trial Registration:**

ISRCTN85759656

## Background

With the ageing of the population, the prevalence of degenerative joint diseases is increasing. Reports show that over a one-year period, 25% of people over 55 years have a persistent episode of knee pain, of whom annually about one in six consult their general practitioner, in both the UK and the Netherlands [1]. The prevalence of painful disabling knee osteoarthritis in people over 55 years is 10% [2], of whom one quarter are severely disabled. In all, over 300,000 Dutch residents currently suffer from knee osteoartritis (OA). Total Knee Arthroplasty (TKA) is a common intervention that can enhance the quality of life for patients with knee OA. Over 7500 TKAs are performed in Dutch hospitals every year. In 2004, more than 160 TKAs were performed at the Maastricht University Hospital.

Adequate and intensive rehabilitation is an important requirement for successful TKA. The primary focus of early rehabilitation is to prepare patients for discharge from the hospital as soon as possible after their operation. Because restricted knee range of motion (RoM) affects functional activities, knee RoM is regarded as one of the primary indicators of a successful TKA. Rapid return of knee RoM accompanied by earlier return to functional activities of daily life was one of the potential effects of the intervention applied in this study.

Continuous passive motion (CPM) is an external motorised device, which enables a joint to move passively throughout a preset arc of motion. Robert Salter introduced the biological concept of CPM in the early 1980s [3-6]. He demonstrated in rabbit knees that CPM enhanced cartilage healing and regeneration compared to prolonged articular rest. Coutts et al [7] first initiated CPM use immediately after TKA. Their rationale was based on Salter's research and the postulate that CPM enhanced collagen tissue healing with better fibre orientation, avoiding cross-linking and thus generating better movement restoration.

CPM has been widely used as an adjunct to physiotherapy (PT) after TKA for the past two decades. However, there is still controversy as to whether it is useful. Various authors recommend CPM [7-15], whereas others [16-23] have found it to be of little value in the rehabilitation of the knee after TKA.

Although several systematic reviews favour the use of CPM in the first rehabilitation phase after surgery [24-26], there still is substantial debate about the total period of CPM application and the duration of individual sessions. A Cochrane review [24] on the topic concluded that use of CPM combined with PT offers beneficial results compared to PT alone in the short-term rehabilitation after TKA. It also suggested, however, that more research was required to assess the differences in CPM effectiveness with different characteristics of application, such as total duration of treatment and intensity of CPM interventions.

Most studies have evaluated effects during the acute in-hospital period. Before the year 2000, discharge from the Maastricht University Hospital after TKA was scheduled approximately 14 days after surgery. Nowadays, most patients are discharged four days after surgery. Since the time spent in hospital after surgery has decreased, continuation of CPM after hospital discharge might be beneficial. Although CPM is now increasingly being administered in the postclinical home situation and is beginning to become part of the usual care programme, proper research into the effectiveness of a prolonged use of CPM at home is still lacking [24,25]. The only study that has been reported [20] compared CPM with PT as a stand-alone therapy, whereas in the study presented here, CPM was added to a standardised programme, adequately reflecting current practice, as orthopaedic surgeons at the hospital and physiotherapists at the hospital and at home currently play an important role in the rehabilitation process for TKA patients.

This study involved the same health care professionals and the same treatment strategies that are currently in use in the Netherlands, but one patient group additionally received CPM at home.

The expected effect of CPM treatment was a quicker restoration of RoM, resulting in improved ADL function during the first three months after surgery. Knee flexion values of 95° and 105° are regarded as RoM benchmarks [27] in the functional recovery after CPM. While 95° of knee flexion allows normal ADL function, 105° of flexion provides the opportunity to ride a bicycle. This is of great advantage both in daily life, at least in the Netherlands, and in the rehabilitation from TKA surgery, because cycling allows patients to move the knee much more. We expected that prolonged use of CPM at home would allow patients to achieve these RoM benchmarks earlier in their recovery process.

The study was conducted among patients with limited RoM at the time of hospital discharge. We chose to include this specific subgroup because we believed that CPM might provide the greatest RoM gain in patients with RoM limitations. Furthermore, several authors have stated that patients with poorer function immediately after surgery may well need more attention [28,29]. About 50% of the patients undergoing one of the 160 TKAs performed annually at the Maastricht University Hospital have less than 80° of RoM four days after surgery and therefore potentially meet the inclusion criteria of the proposed study.

### Objective

Continuous passive motion (CPM) has proved to increase the amount of knee flexion for knee patients in the acute hospital setting (5–10 days). The primary purpose of this randomised controlled trial was to establish whether there is additional longer-term benefit of continuing CPM after hospital discharge.

### Research question

What is the effect on range of motion and functional status of prolonged use of a continuous passive motion device at home in addition to PT, compared to PT alone, in patients with limited flexion range of motion (less than 80°) of the knee at discharge from the hospital after total knee arthroplasty?

## Methods

### Study design

A randomised controlled trial, with blinded treatment allocation, assessment and analysis, was carried out, with local medical ethics committee approval, to assess the added value of prolonged CPM use at home, using function and mobility as the main outcomes.

### Participants

Patients scheduled for unilateral primary TKA between April 1^st ^2005 and June 30th 2006 in the 'Arthrose kliniek Maastricht' (Maastricht osteoarthritis clinic) programme at the Maastricht University Hospital, the Netherlands, were invited to participate in the study. Subjects were considered eligible if they had less than 80° of RoM 4 days after surgery, were able to understand and speak Dutch, were not suffering from mental disabilities and were resident within the 'Maastricht Heuvelland' region. Patients were excluded if they needed to stay in hospital for more than five days after surgery or showed relevant co-morbidity influencing mobility (e.g. claudication, other prosthesis) or were operated upon by minimally invasive surgery. Patients older than 80 years were also excluded. Eligible patients were contacted one week before the planned surgery, and were randomised into two groups after signing an informed consent form.

### Randomisation

Blocked and concealed randomisation with a block size of four ensured equal distribution of patients over the two treatment groups. Groups were prestratified on preoperative flexion mobility of the knee.

### Interventions

During the in-hospital period, all patients received a standardised PT programme, involving 20 minutes of PT and four hours of CPM use daily for four days. CPM was already applied in the recovery room. Nurses installed the CPM device following standardised procedures. CPM was used for two consecutive hours, twice daily. One session was performed in the evening in order to avoid interfering with other daytime medical and rehabilitation activities. After 5 minutes of warm-up, RoM was set as tolerated by the patients. A description of the treatment protocol after total knee surgery is available on the website of the PT department of the Maastricht University Hospital [30].

At the end of the in-hospital period, all patients were randomly assigned to one of the following groups: a control group which received semi-standardised regular PT and an experimental group which received the same PT intervention in combination with two extra weeks of CPM treatment for four hours daily. CPM was administered in the same fashion as during hospital stay. Patients were able to increase RoM by themselves. They kept a patient diary in which they wrote the daily RoM increments as well as the content and duration of their PT sessions, medication use and pain perceived during the day and while on the CPM machine.

From day 18 onward, all patients received regular PT treatment until patients and therapists were satisfied with their overall functioning.

The post-clinical PT was standardised in terms of treatment objectives. All patients received treatment consisting of active and passive mobilisation of the knee joint, active strengthening of the m. quadriceps, and training of ADL functions (gait, sit to stand and stair climbing). Mean treatment session duration was 30 minutes, mean total of treatment sessions

### Outcome assessment

After collection of baseline variables before surgery and at hospital discharge, outcome measures were assessed at 17 days, six weeks and three months after surgery, during normal routine assessments at the orthopaedic clinic (Table 1) or by visiting the patients at home. The outcome assessor was blinded for the treatment procedure.

**Table 1:** Timing of the outcome assessment

	T0 1 WEEK PRIOR TO SURGERY	T1 END OF CLINICAL PHASE	T2 17 DAYS AFTER SURGERY	T3 6 WEEKS AFTER SURGERY	T4 12 WEEKS AFTER SURGERY
Range of motion	X	X	X	X	X
Knee Society Score	X	X	X	X	X
WOMAC	X		X	X	X
Perceived effect			X	X	X
Pain medication		X	X	X	X
Satisfaction with treatment			X	X	X
Satisfaction with results			X	X	X
Adherence			X		
Quantity, duration and kind of treatment			X	X	X

Primary outcome measures were:

1. functional status, using the WOMAC function score [31,32] and the Knee Society Score [33]; and

2. range of motion, assessed with a long-arm goniometer [34].

Secondary outcome measures were:

a. perceived effect, using a seven-point Likert scale;

b. postoperative medication use (amount; type being standardised);

c. satisfaction with treatment, on an 11-point Likert scale;

d. satisfaction with treatment result, on an 11-point Likert scale;

e. adherence to treatment protocols and use of CPM (in hours);

f. quantity, duration and nature of PT intervention.

**Figure 1:**
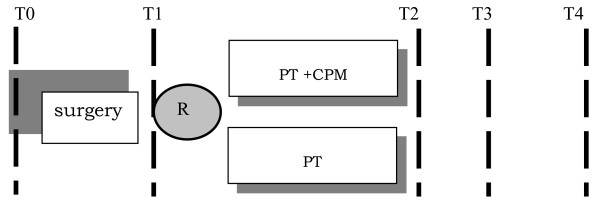
**Study design and outcome assessment**. T0 baseline assessment, one week before surgery, T1 assessment 4 days after surgery, T2 assessment 17 days after surgery, T3 assessment 6 weeks after surgery T4 assessment 3 months after surgery, R = randomisation.

The study design is depicted in Figure 1.

The first primary endpoint of the study was on day 17 after surgery, which was the time when the experimental treatment stopped and short-term effects were measured.

The second endpoint was at three months after surgery.

Table 1 shows the timing of the outcome assessment

RoM was measured actively as well as passively using a large goniometer following the method described by Brosseau [34]. The intraobserver reliability of this method for knee flexion is 0.99, that for active extension 0.97, and the criterion validity for knee flexion is 0.98, that for extension 0.42 [34].

Functional status was measured using two scales, the joint specific Knee Society Scale (KSS) [33] and the disease-specific Western Ontario and McMaster University Osteoarthritis index (WOMAC) [31].

The KSS is concise and easy to use. It represents a clear attempt to separate knee function from overall patient functional status. Bach et al [35] reported that the reproducibility of the knee score is poor, whilst the function score shows good reproducibility. The construct validity of the KSS is good [36].

The WOMAC Osteoarthritis Index is a disease-specific questionnaire developed specifically for people with osteoarthritis of the hip and knee. It is a self-administered, 3-dimension, 24-item instrument. The three dimensions of the WOMAC are pain, stiffness, and physical function. Scoring of the WOMAC ranges from 'none' to 'extreme'. Scale sum scores have been standardised (0–100), with high values indicating less pain or better physical functioning [32]. The WOMAC questionnaire is generally acknowledged to have good validity, reliability and responsiveness. We used the Dutch version of WOMAC [32].

At follow-up, patients were asked to judge the effect of the surgery on a seven-point Likert scale ranging from 'worse than ever' status to 'completely recovered' [37].

Postoperative medication use and adherence to the treatment protocols were measured using a patient diary.

The primary effect measurement was scheduled for the 17th day after surgery, while follow-up measurements were scheduled at six weeks and three months after surgery.

### Power analyses

The number of subjects required to achieve statistical significance was determined by means of a power analysis. We assumed that a difference of more than 5° of knee flexion mobility (SD 8°) at the end of the CPM application would be clinically relevant. With an alpha of 0.05, and a power of 80%, we needed 28 patients per group to prove this.

### Data analysis

Data was stored and analysed with SPSS-12.0 After checks for missing values and normality, linear regression techniques were applied by a blinded analyst using the 'intention-to-treat' principle. Primary and secondary outcome measures were reported for the in-hospital and home situations, and for six-week and three-month follow-up. Means and 95% confidence intervals were calculated.

The primary research question was tested using Student's t-tests with a p-value of 0.05 being regarded as statistically significant.

## Results

Of the 147 patients who were scheduled for surgery between April 1^st ^2005 and June 30th 2006, 60 were included in the study. Thirty-five patients were not considered eligible, for the following reasons. Five patients were not residents of the Maastricht region, 10 patients had relevant co-morbidity or had RA as an underlying problem and 20 patients were older than 80 years. Another 52 patients were excluded, for the following reasons: 32 were excluded because their active knee RoM was over 80° at T1; eight patients needed to stay in hospital for more than five days after surgery and six were operated upon using minimally invasive surgery. In four cases, surgery was more extensive. Two patients refused to participate because of bad experiences with CPM treatment during the in-hospital period.

The baseline characteristics of the patients in the two groups were similar in terms of clinical and demographic characteristics.

All Patients were operated by one of two orthopaedic surgeons. One surgeon operated on 40 patients, equally divided over both treatment groups. The other surgeon performed the surgery in 20 cases, also equally divided over both groups. Cemented Scorpio ps knee prosthesis were used and the patella was resurfaced in all cases. Mean operation time was 111 minutes, 109 (sd 20.7) in the CPM+ PT group and 113 (sd 22.0) in the PT group.

Although we prestratified on preoperative active RoM (strata > 100° <), mean preoperative flexion RoM was slightly higher in the CPM group. At T1, RoM was similar in both groups. Both groups also showed comparable levels of functional ability (Tables 2 and 3).

**Table 2:** Subject characteristics and preoperative assessments of RoM and functional status

T0	PT + CPM N = 30		PT N = 30	
		
Gender % female	60		70	
	Mean	Sd	Mean	Sd
Age	64.1	8.1	65	9.1
Active RoM before surgery (flexion to extension)	104.9	13.8	100.7	13.2
Passive RoM before surgery	110.2	14.0	105.9	13.8
Active extension	4.8	4.6	6.2	5.0
Active flexion	109.7	12.1	106.9	12.3
Passive extension	3.3	4.3	4.7	4.3
Passive flexion	113.5	12.4	110.6	11.8
KSS knee	53.5	16.4	47.5	15.3
KSS function	61.5	10.6	52.3	18.7
WOMAC score	54.8	17.7	51.2	13.6
Pain subscale	10.6	4.7	10.5	3.1
Stiffness subscale	4. 0	1.9	3.8	2.1
Difficulty subscale	40. 2	13.2	36.9	11.7

**Table 3:** Baseline measurement (T1) of RoM and KSS

T1	PT + CPM N = 30		PT N = 30	
		
Active RoM at T1	66.1	8.9	66.6	6.8
Passive RoM at T1	71.1	8.4	72.5	5.7
Active extension	8.9	4.4	8.1	4.4
Active flexion	75	6.9	74.7	4.5
Passive extension	6.7	4.1	6.1	4.2
Passive flexion	77.8	7.1	78.6	3.8
Knee society knee score at T1	55.9	17	50.0	17.6
Knee society function score at T1	25.8	14	26	13.5

T1 = day of discharge from the hospital, i.e. day 4 after surgery

### Primary outcome measures

#### Range of motion

A trend in favour of prolonged CPM use was found at the end of the treatment period. In terms of total RoM, the CPM group achieved 5° more RoM than the PT group (Table 4). RoM in the CPM group improved 6° more during the active treatment period (17. 7° over 11. 6°).

**Table 4:** Primary outcome measurements on day 17

T2 ROM	PT + CPM N = 30		PT N = 30		P VALUE	95% CI
	Mean	sd	Mean	sd		
			
Active RoM at T2	83.6	11.4	78.6	8.7	0.06	-0.2 – 10.3
Passive RoM at T2	88.7	10.4	84.0	9.9	0.07	-0.5 – 10.0
Delta active RoM T1-T2	17.5	13.4	11.9	9.8	0.07	-0.5 – 11.6
Delta passive RoM T1-T2	17.6	12.9	11.6	9.4	0.04	0.02 – 11.9
Extension active	6.3	3.9	8.1	4.8	0.11	-4.1 – 0.4
Extension passive	4.3	3.1	5.7	4.6	0.17	-3.4 – 0.6
Flexion active	89.9	9.1	86.7	8.5	0.16	-1.4 – 7.8
Flexion passive	93	8.8	89.7	9.6	0.17	-1.4 – 8.1
KSS knee score	67.6	19.6	67.3	14.9	0.94	-8.7 – 9.3
KSS function score	43	14.6	39.8	21.1	0.50	-4.7 – 11.3
WOMAC score	69.9	15.9	65.4	16.4	0.28	-3.9 – 12.9
WOMAC pain	15.8	4.7	15.3	4.1	0.60	-2.9 – 1.7
WOMAC stiffness	5.0	1.8	4.8	1.6	0.66	-0.7 – 1.1
WOMAC difficulty	49.1	11.9	45.3	12.3	0.23	-2.5 – 10.1

T2 = 17 days after surgery

At follow-up, these differences had faded. At six weeks and three months, we could not detect any difference in RoM between the two treatment strategies (Figure 3). In terms of the number of patients achieving the flexion RoM benchmarks of 95° and 105°, no differences were found between the two groups at any of the outcome measurements (Table 5).

**Figure 2:**
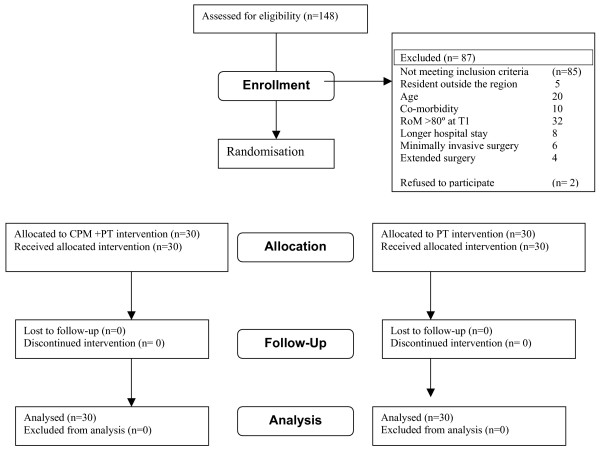
Flowchart of subjects through the trial.

**Figure 3:**
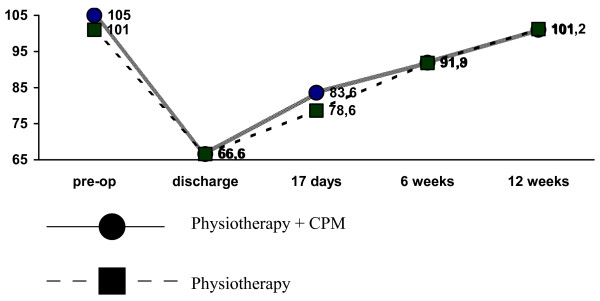
Progress of active RoM through the trial period.

**Table 5:** Number of patients reaching flexion benchmarks at T2 and T3

	T2 17 DAYS AFTER SURGERY				T3 6 WEEKS AFTER SURGERY			
		
Group	< 95° >		< 105° >		< 95° >		< 105° >	
				
CPM + PT	25	5	27	3	10	20	20	10
PT	24	6	28	2	10	20	22	8

#### Functional status

Although the experimental group scored slightly better on the KSS functional status score and the WOMAC functional difficulty score, no significant differences were found between the two groups, neither at day 17 nor at either of the follow-up measurements (Tables 4 and 6).

**Table 6:** Six-week and three-month follow-up

	6 WEEKS						3 MONTHS					
		
	PT+CPM n = 30		Pt n = 30		P value	95ci	PT+CPM n = 30		Pt n = 30		P value	95ci
	Mean	sd	Mean	sd			Mean	sd	Mean	sd		
						
Active RoM	91.9	13.6	91.8	14.3	0.98	-7. – 7.3	100.9	4.8	102	4.3	0.78	-8.8 – 6.6
Passive RoM	96.9	13.4	95.5	14.2	0.69	-5. – 8.5	106.1	14.1	106.2	3.7	0.98	-7.5 – 7.3
Extension active	6.3	4.0	6.9	5.4	0.63	-3.0 – 1.8	4.8	3.9	4.3	4.7	0.61	-1.7 – 2.9
Flexion active	98.2	11.7	98.7	11.2	0.87	-6. – 5.4	105.7	2.5	106.2	0.6	0.99	-6.7 – 5.6
KSS knee score	77.3	14.9	73.6	13.8	0.33	-3.8 -11.1	80.4	5.3	78.8	9.2	0.72	-7.6 – 10.8
KSS function score	59.8	10.0	56.2	15.8	0.29	-3.2 – 0.5	72.4	11.4	65.5	2.1	0.15	-2.6 – 16.3
WOMAC score	75	13.6	74.5	16.1	0.89	-7.2 – 8.2	80.5	7.7	82.8	0.5	0.56	-10.5 – 5.5
WOMAC pain	16.0	3.7	16.6	4.0	0.53	-2.6 – 1.4	17.3	3.8	17.5	.9	0.83	-2.3 – 1.8
WOMAC stiffness	5.4	1.5	4.8	1.5	0.13	-0.2 – 1.4	5.5	1.4	5.3	1.6	0.49	-0.5 – 1.0
WOMAC difficulty	53.0	9.5	52.7	12.0	0.92	-5.3 – 5.9	57.6	4.2	58.6	8.4	0.74	-7.3 – 5.2
Perceived effect	2.3	0.76	2.4	0.85	0.87	-0.45 -.38	2.1	0.7	2.4	1.0	0.16	-0.73 – 0.14
Satisfaction with treatment	8.3	0.88	8.7	0.84	0.76	-0.85 -0.1	8.6	0.77	8.5	0.88	0.70	-0.35 – 0.52
Satisfaction with results	8.1	1.0	8.4	1.0	0.21	-0.85 -.18	8.6	0.8	8.2	1.5	0.20	-0.22 – 1.0

#### Secondary outcome

No significant differences were found on any of the secondary outcome measures at any single outcome measurement. Treatment content was similar in both groups, as were the number of treatment sessions and the time spent on individual sessions. As early as day 17, the majority of the patients scored their status (perceived effect) as 'better' compared to the situation before surgery. Patients were generally satisfied with their individual treatment and with treatment results (Table 7).

**Table 7:** Secondary outcome assessment on day 17

T2 ROM	PT + CPM N = 30		PT N = 30		P value	95% CI
			
	MEAN	SD	MEAN	SD		
Number of treatment sessions	4.8	1.5	5.1	1.4	0.43	-1.05 – 0.44
Duration per session	29.5	5.5	28.4	5.9	0.49	-2.0 – 4.13
Perceived effect	3.2	1.5	3.0	1.5	0.55	-0.5 – 1.0
Satisfaction with treatment	8.2	0.8	8.5	0.7	0.37	-1.0 – -0.1
Satisfaction with results	7.4	1.4	7.8	1.5	0.35	-1.2 – 0.3
Adherence	97%		100%			

Adherence to the CPM intervention was very high: 29 of the 30 patients followed the prescribed regimen. One patient reduced CPM use to two hours from day 10 onwards, because her knee RoM was over 105°, and she felt uncomfortable with four hours of CPM use when reaching high degrees of flexion.

#### Complications

One subject in each group had to undergo knee manipulation under anaesthesia between six weeks and three months after surgery.

Secondary analyses on the subgroup of the patients having less than 68° (0–50 percentile) of active RoM at discharge showed that these patients benefited more from CPM treatment. They improved 11° more in active RoM(22° versus 11° in the PT group) during the active treatment fortnight. However, these differences in range of motion benefit had also faded at six-week follow-up.

## Discussion

The present study demonstrated improvement in total RoM at the end of the prolonged period of CPM use. However, this did not translate into any functional benefits. Our findings thus suggest that, although CPM produces benefits in knee RoM in the short term, it does not result in additional RoM in the longer term, nor in any functional gain. Since we did not find any difference in the numbers of patients achieving the clinically important benchmarks of 95° and 105°, neither at the end of the treatment period nor at follow-up, it is doubtful whether the additional degrees of RoM are of clinical importance.

Although our study population was a selection of patients with limited RoM at discharge, our results confirm those presented in systematic reviews [24,25], implying that patients with limited RoM exhibit comparable improvements to the basic population of patients after TKA. Our hypothesis that this group might benefit more from the CPM application was not supported. Our long-term results were comparable to those reported by others [12,15,38]. Kumar et al [10] and Leach et al [23] reported greater RoM at six-week follow-up. A possible explanation might be that they included patients, regardless of RoM and therefore found somewhat better RoM.

Like previous researchers [9,15,16,39], we did not detect any differences in functional status between the groups. The addition of CPM did not seem to lead to measurable functional benefits. Denis et al [16] surmised that subjects who received additional CPM could even have poorer functional abilities, because they remained inactive during the CPM interventions. We did not detect a decrease in functional activities in the CPM group. A large proportion of all subjects, regardless of research group allocation, reported functional gains on all outcome measurements. Patients with limited RoM in the early stages of recovery seemed to consistently improve over time.

We chose total RoM as the outcome instead of focusing on flexion RoM because several authors [8,40-42] have already reported on adverse effects of CPM application on extension range. Although extension RoM in our patients was limited in the short term after TKA, we did not detect any difference between the two groups. In fact, we found slightly better extension RoM in the CPM group. Our extension deficits were comparable to those already reported by others [9,10,16,22].

We found small effects on range of motion at the end of the active treatment period, which faded during four weeks of follow-up in which the patients received regular PT treatment. This suggests that although adding 14 days of CPM is beneficial for short-term RoM, the improvement does not last. An alternative hypothesis might be that CPM treatment should be maintained for an even longer period for effects to take root. Our study results do not rule out this hypothesis.

### Study limitations

Milne et al [24] suggested in their review that future research should focus on the effects of different characteristics of application, such as total duration of treatment and intensity of CPM interventions, and on effects in different populations. We chose to include patients with limited range of motion at the end of the in-hospital period, postulating that these patients might benefit most from extra CPM treatment. Perhaps our choice of target population was wrong. In any case, one should keep in mind that our conclusions are limited to comparable populations, post-clinical PT treatment and CPM application protocols similar to those described in our clinical trial. This may be obvious, but as several authors [43,44] have already pointed out, the treatment approaches used after TKA vary greatly among rehabilitation providers. The external validity of our study may therefore be limited if protocols differ greatly.

We protocolised the post-clinical PT in terms of treatment objectives, leaving individual therapists some leeway to specify these objectives at their own discretion. This may have led to differences in treatment content between individual therapists, which might have affected the contrast between the two interventions. However, we did not detect any major differences in PT treatment from the patient diaries in which therapists wrote down the content of the individual treatment sessions during the active CPM treatment period.

CPM treatment in the home situation was a self-management treatment. Patients received the CPM machine, which was adjusted to their individual needs and characteristics. Patients were instructed how to use it and for how long. During the next fortnight, patients managed treatment on their own or with the help of their spouses. Although they were instructed to call the researcher if they had any problems with the machine, this only occurred twice. Although analysis of the patient diaries in which they wrote daily CPM increments and usage time did not indicate any misuse in CPM application, and although self-management is usual care for at-home CPM use in the Netherlands, it may have led to sub-maximal use of the CPM device.

Our study has many characteristics that contribute to the validity of the results, such as comparability at baseline, adherence to interventions and the use of one outcome assessor to minimise noise due to problems of interobserver reproducibility.

## Conclusion

Although our results indicate that prolonged CPM use has a short-term effect on RoM, standard implementation of prolonged CPM use in patients with limited RoM at hospital discharge should be reconsidered, since neither long-term effects nor transfer to better functional performance was detected.

## Competing interests

The authors declare that they have no competing interests.

## Authors' contributions

AFL participated in the design of the study and participated in the assessments and follow-ups, statistical analyses and writing. YC and EW participated in design and in the assessments and follow-ups. MvS, RG, PvdB and RdB participated in the design. All authors have read and approved the final manuscript.

## Pre-publication history

The pre-publication history for this paper can be accessed here:


